# Supplementary material for: Blood Phytosterol Concentration and Genetic Variant Associations in a Sample Population
Source: Nutrients. 2024 Apr 5;16(7):1067. doi: 10.3390/nu16071067 (PMC11013666; doi:10.3390/nu16071067)
Supplement: Supplementary file 1 [file nutrients-16-01067-s001.zip › nutrients-2882460-supplementary.pdf]

Supplementary material

S1. Plasma Sterol levels and variants

PS Fraction	rs4148217			*p**
	CC (n=122)	CA (n=58)	AA (n=5)	
**Total PS** (µg/mL)	2.08±1.59	1.65±1.22	1.80±1.36	0.19
**Campesterol** (µg/mL)	0.90±0.91	0.74±0.91	0.97±1.08	0.51
**Sitosterol** (µg/mL)	0.91±0.74	0.70±0.45	0.64±0.31	0.12
**Sitostanol** (µg/mL) *10	0.25±0.19	0.22±0.16	0.17±0.13	0.27
**Lanosterol** (µg/mL) *10	0.95±1.02	0.76±0.69	0.82±0.73	0.41
**Stigmasterol** (µg/mL) *10	1.05±1.43	1.00±0.91	0.64±0.39	0.77
**Total Cholesterol** (mg/dL)	104.93±60.62	97.93±51.14	74.75±54.86	0.43

PS Fraction	rs4245791			*p**
	CC (n=19)	TC (n=79)	TT (n=87)	
**Total PS** (µg/mL)	2.37±2.06	2.00±1.63	1.78±1.16	0.26
**Campesterol** (µg/mL)	1.09±1.12	0.95±1.12	0.71±0.58	0.12
**Sitosterol** (µg/mL)	1.02±0.93	0.82±0.64	0.81±0.62	0.42
**Sitostanol** (µg/mL) *10	0.26±0.22	0.22±0.16	0.25±0.19	0.50
**Lanosterol** (µg/mL) *10	0.99±1.03	0.90±1.04	0.85±0.79	0.84
**Stigmasterol** (µg/mL) *10	1.07±1.09	1.01±1.51	1.02±1.06	0.99
**Total Cholesterol** (mg/dL)	104.22±69.53	106.01±62.13	97.71±50.62	0.64

PS Fraction	rs657152			P*
	GG (n=66)	GT (n=90)	TT (n=29)	
**Total PS** (µg/mL)	1.71±1.27	2.11±1.71	1.93±1.12	0.26
**Campesterol** (µg/mL)	0.67±0.58	0.98±1.15	0.84±0.53	0.11
**Sitosterol** (µg/mL)	0.76±0.65	0.88±0.69	0.87±0.62	0.49
**Sitostanol** (µg/mL) *10	0.22±0.18	0.25±0.19	0.24±0.18	0.57
**Lanosterol** (µg/mL) *10	0.83±0.78	0.97±1.11	0.75±0.51	0.45
**Stigmasterol** (µg/mL) *10	1.17±1.81	0.96±0.85	0.87±0.77	0.48
**Total Cholesterol** (mg/dL)	103.92±54.33	99.36±64.06	105.31±43.56	0.84

PS Fraction	rs11887534		*p**
	GC (n=27)	GG (n=158)	
**Total PS** (µg/mL)	1.86±1.87	1.95±1.42	0.77
**Campesterol** (µg/mL)	0.92±1.35	0.84±0.82	0.68
**Sitosterol** (µg/mL)	0.71±0.52	0.85±0.68	0.31
**Sitostanol** (µg/mL) *10	0.21±0.14	0.24±0.19	0.36
**Lanosterol** (µg/mL) *10	1.03±0.90	0.86±0.93	0.40
**Stigmasterol** (µg/mL) *10	0.69±0.36	1.08±1.36	0.00
**Total Cholesterol** (mg/dL)	98.97±50.95	102.43±58.85	0.78

PS Fraction	rs41360247		*P**
	TC (n=26)	TT (n=159)	
**Total PS** (µg/mL)	1.86±1.90	1.95±1.41	0.78
**Campesterol** (µg/mL)	0.91±1.38	0.84±0.81	0.71
**Sitosterol** (µg/mL)	0.68±0.54	0.86±0.68	0.19
**Sitostanol** (µg/mL) *10	0.21±0.14	0.24±0.19	0.31
**Lanosterol** (µg/mL) *10	1.31±1.40	0.82±0.81	0.09
**Stigmasterol** (µg/mL) *10	0.85±0.63	1.05±1.34	0.45
**Total Cholesterol** (mg/dL)	94.61±52.44	103.12±58.52	0.49

* P by F Snedecor

S2a. Association between plasma phytosterols and categorical variables (univariant analysis)

Variable (n)	Mean±SD	*p*
Total phytosterols (μg/mL)		
Sex		0.12
Women (n=115)	2.06±1.70	
Men (n=70)	1.74±1.03	
Pastries		0.08
No consumption (n=94)	2.12±1.70	
1-3 times a week (n=78)	1.84±1.25	
>3 times a week (n=13)	1.18±0.59	
Nuts		0.82
No consumption (n=58)	1.84±1.52	
1-3 times a week (n=99)	1.97±1.56	
>3 times a week (n=28)	2.03±1.12	
Sausages		0.36
No consumption (n=24)	1.74±1.00	
1-3 times a week (n=103)	2.08±1.77	
>3 times a week (n=58)	1.77±1.01	
**Cholesterol-lowering treatment**		0.89
No (n=169)	1.94±1.51	
Yes (n=16)	1.89±1.21	
**Vitamins**		0.98
No (n=173)	1.93±1.51	
Yes (n=12)	1.95±1.05	
**PS-enriched supplements**		0.21
No (n=177)	1.91±1.48	
Yes (n=8)	2.58±1.42	
**Vegetarian-vegan**		0.90
No (n=176)	1.94±1.50	
Yes (n=9)	2.00±1.24	
Sitosterol (μg/mL)		
Sex		**0.01**
Women (n=115)	0.93±0.75	
Men (n=70)	0.68±0.46	
Pastries		**0.04**
No consumption (n=94)	0.89±0.70	
1-3 times a week (n=78)	0.84±0.64	
>3 times a week (n=13)	0.40±0.27	
Nuts		0.48
No consumption (n=58)	0.75±0.59	
1-3 times a week (n=99)	0.88±0.74	

>3 times a week (n=28)	0.84±0.52	
Sausages		0.70
No consumption (n=24)	0.86±0.57	
1-3 times a week (n=103)	0.86±0.76	
>3 times a week (n=58)	0.77±0.50	
**Cholesterol-lowering treatment**		0.16
No (n=169)	0.86±0.68	
Yes (n=16)	0.61±0.48	
**Vitamins**		0.65
No (n=173)	0.83±0.67	
Yes (n=12)	0.92±0.60	
**PS-enriched supplements**		0.10
No (n=177)	0.82±0.65	
Yes (n=8)	1.21±0.79	
**Vegetarian-vegan**		0.58
No (n=176)	0.83±0.66	
Yes (n=9)	0.96±0.70	
Campesterol (µg/ml)		
Sex		0.59
Women (n=115)	0.88±1.02	
Men (n=70)	0.81±0.69	
Pastries		0.11
No consumption (n=94)	0.98±1.13	
1-3 times a week (n=78)	0.74±0.60	
>3 times a week (n=13)	0.57±0.34	
Nuts		0.85
No consumption (n=58)	0.84±1.08	
1-3 times a week (n=99)	0.83±0.85	
>3 times a week (n=28)	0.94±0.73	
Sausages		0.22
No consumption (n=24)	0.65±0.41	
1-3 times a week (n=103)	0.95±1.07	
>3 times a week (n=58)	0.76±0.72	
**Cholesterol-lowering treatment**		0.29
No (n=169)	0.83±0.92	
Yes (n=16)	1.08±0.75	
**Vitamins**		0.86
No (n=173)	0.85±0.93	
Yes (n=12)	0.81±0.47	
**PS-enriched supplements**		0.38
No (n=177)	0.84±0.92	

Yes (n=8)	1.13±0.66	
**Vegetarian-vegan**		0.70
No (n=176)	0.86±0.93	
Yes (n=9)	0.74±0.48	
	Sitostanol (µg/ml)	
Sex		**<0.01**
Women (n=115)	0.03±0.02	
Men (n=70)	0.02±0.01	
Pastries		**0.03**
No consumption (n=94)	0.02±0.02	
1-3 times a week (n=78)	0.03±0.02	
>3 times a week (n=13)	0.01±0.01	
Nuts		0.49
No consumption (n=58)	0.02±0.01	
1-3 times a week (n=99)	0.03±0.02	
>3 times a week (n=28)	0.02±0.02	
Sausages		0.27
No consumption (n=24)	0.03±0.01	
1-3 times a week (n=103)	0.02±0.02	
>3 times a week (n=58)	0.02±0.02	
**Cholesterol-lowering treatment**		**<0.01**
No (n=169)	0.03±0.02	
Yes (n=16)	0.01±0.01	
**Vitamins**		0.84
No (n=173)	0.02±0.02	
Yes (n=12)	0.02±0.02	
**PS-enriched supplements**		0.23
No (n=177)	0.02±0.02	
Yes (n=8)	0.03±0.02	
**Vegetarian-vegan**		0.12
No (n=176)	0.02±0.02	
Yes (n=9)	0.03±0.02	
	Lanosterol (µg/ml)	
Sex		**0.01**
Women (n=115)	0.07±0.07	
Men (n=70)	0.12±0.11	
Pastries		0.08
No consumption (n=94)	0.10±0.10	
1-3 times a week (n=78)	0.07±0.08	
>3 times a week (n=13)	0.12±0.11	

Nuts		0.15
No consumption (n=58)	0.10±0.12	
1-3 times a week (n=99)	0.08±0.06	
>3 times a week (n=28)	0.11±0.10	
Sausages		0.54
No consumption (n=24)	0.07±0.06	
1-3 times a week (n=103)	0.09±0.09	
>3 times a week (n=58)	0.10±0.10	
**Cholesterol-lowering treatment**		0.43
No (n=169)	0.09±0.09	
Yes (n=16)	0.11±0.08	
**Vitamins**		0.68
No (n=173)	0.09±0.09	
Yes (n=12)	0.08±0.07	
**PS-enriched supplements**		0.67
No (n=177)	0.09±0.09	
Yes (n=8)	0.10±0.12	
**Vegetarian-vegan**		0.76
No (n=176)	0.09±0.09	
Yes (n=9)	0.08±0.06	
Stigmasterol (µg/ml)		
Sex		0.50
Women (n=115)	0.11±0.14	
Men (n=70)	0.09±0.10	
Pastries		0.19
No consumption (n=94)	0.10±0.10	
1-3 times a week (n=78)	0.12±0.16	
>3 times a week (n=13)	0.05±0.03	
Nuts		0.66
No consumption (n=58)	0.09±0.07	
1-3 times a week (n=99)	0.11±0.16	
>3 times a week (n=28)	0.09±0.06	
Sausages		0.63
No consumption (n=24)	0.09±0.05	
1-3 times a week (n=103)	0.11±0.16	
>3 times a week (n=58)	0.09±0.08	
**Cholesterol-lowering treatment**		0.08
No (n=169)	0.11±0.13	
Yes (n=16)	0.05±0.04	
**Vitamins**		0.95
No (n=173)	0.10±0.13	
Yes (n=12)	0.10±0.10	
**PS-enriched supplements**		0.60

No (n=177)	0.10±0.13	
Yes (n=8)	0.08±0.04	
**Vegetarian-vegan**		0.34
No (n=176)	0.10±0.12	
Yes (n=9)	0.17±0.20	

S2b. Association between plasma phytosterols and continuous variables (univariant analysis)

**Variable (n)**	**B (95% CI)**	***p*** **(R^2)**
	Total phytosterols (µg/mL)	
Age (years)	0.008 (-0.003 – 0.020)	0.154 **(**0.011)
BMI (kg/m²)	-0.041 (-0.091 – 0.009)	0.108 (0.014)
**α-Tocopherol** (µmol/L)	0.011 (-0.023– 0.046)	0.519 (0.002)
FI	-0.071 (-0.258 – 0.116)	0.455 (0.003)
VII	0.081 (-0.082 – 0.244)	0.328 (0.005)
**Creatinine** (µmol/L)	-0.010 (-0.023 – 0.003)	0.118 (0.013)
AP (µkat/L)	0.448 (-0.329 – 1.226)	0.257 (0.007)
ALT (µkat/L)	-0.421 (-2.09 – 1.251)	0.620 (0.001)
	Sitosterol (µg/mL)	
Age (years)	-0.005 (-0.010 – 0.000)	**0.050** (0.021)
BMI (kg/m²)	-0.035 (-0.057 – (-0.013)	**0.002** (0.052)
**α-Tocopherol** (µmol/L)	0.008 (-0.008 – 0.023)	0.319 (0.005)
FI	-0.057 (-0.140 – 0.027)	0.181 (0.010)
VII	0.063 (-0.010 – 0.135)	0.090 (0.016)
**Creatinine** (µmol/L)	-0.003 (-0.009 – 0.003)	0.300 (0.006)
AP (µkat/L)	-0.076 (-0.424 – 0.273)	0.669 (0.001)
ALT (µkat/L)	-0.308 (-1.054 – 0.438)	0.416 (0.004)
	Campesterol (µg/ml)	
Age (years)	0.012 (0.005 – 0.019)	**0.001** (0.059)
BMI (kg/m²)	-0.010 (-0.041 – 0.021)	0.521 (0.002)
**α-Tocopherol** (µmol/L) **¥**	-0.003 (-0.210 – 2.100)	0.998 (0.000)
FI	-0.028 (-0.143 – 0.086)	0.626 (0.001)
**VII**	0.017 (-0.083 – 0.117)	0.739 (0.001)
**Creatinine** (µmol/L)	-0.007 (-0.015 – 0.000)	0.062 (0.019)
AP (µkat/L)	0.429 (-0.046 – 0.903)	0.076 (0.017)
ALT (µkat/L)	-0.255 (-1.279 – 0.769)	0.624 (0.001)
	Sitostanol (µg/ml)	
Age (years)	0.000 (0.000 – 0.000)	**<0.001** (0.095)
BMI (kg/m²)	-0.001 (-0.002 – 0.000)	**<0.001** (0.066)
**α-Tocopherol** (µmol/L) **¥**	0.008 (0.000 – 0.001)	0.713 (0.001)
FI	-0.001 (-0.003 – 0.001)	0.467 (0.003)
VII	0.002 (0.000 – 0.004)	**0.032** (0.025)

**Creatinine** (µmol/L) **¥**	-0.008 (-0.000 – 0.000)	0.315 (0.006)
AP (µkat/L)	-0.009 (-0.019 – 0.000)	**0.052** (0.020)
ALT (µkat/L)	-0.011 (-0.032 – 0.009)	0.284 (0.006)
	Lanosterol (µg/ml)	
Age (years)	0.002 (0.002 – 0.003)	**<0.001** (0.229)
BMI (kg/m^2)	0.007 (0.004 – 0.010)	**<0.001** (0.095)
**α-Tocopherol** (µmol/L)	0.001 (-0.001 – 0.003)	0.458 (0.003)
FI	0.006 (-0.006 – 0.018)	0.321 (0.005)
VII	-0.001 (-0.011 – 0.009)	0.847 (0.000)
**Creatinine** (µmol/L) **¥**	0.008 (-0.100 – 0.100)	0.843 (0.000)
AP (µkat/L)	0.056 (0.008 – 0.105)	**0.021** (0.029)
ALT (µkat/L)	0.062 (-0.042 – 0.166)	0.243 (0.007)
	Stigmasterol (µg/ml)	
Age (years)	0.000 (-0.001 – 0.001)	0.357 (0.005)
BMI (kg/m^2)	0.000 (-0.004 – 0.005)	0.865 (0.000)
**α-Tocopherol** (µmol/L)	0.002 (-0.001 – 0.005)	0.151 (0.011)
FI	-0.001 (-0.017 – 0.015)	0.887 (0.000)
VII	0.003 (-0.011 – 0.017)	0.681 (0.001)
**Creatinine** (µmol/L)	0.000 (-0.001 – 0.001)	0.794 (0.000)
AP (µkat/L)	0.012 (-0.054 – 0.079)	0.717 (0.001)
ALT (µkat/L)	0.123 (-0.019 – 0.265)	0.089 (0.016)

FI: Fat intake index; VII: vegetal intake index; AP: alkaline phosphatase; ALT: alanine transaminase
*¥B (95% CI)*100*

S3. Bonferroni simultaneous multiple comparison test: Total phystosterol, campesterol and sitosterol for each variant (rs424579, rs4148217PS and rs6577152)

**Variants**	**Total PS**		**Sitosterol**		**Campesterol**	
	Mean differences	*P*	Mean differences	*p*	Mean differences	*p*
rs4245791_TT vs. rs4245791_CC	-0.57	0.321	-0.17	0.683	-0.47	0.077
rs4245791_TC vs. rs4245791_CC	-0.41	0.574	-0.23	0.345	-0.20	0.722
rs4148217_AA vs. rs4148217_CC	0.10	1.000	-0.17	0.891	0.40	0.614
rs4148217_AC vs. rs4148217_CC	-0.39	0.149	-0.19	0.059	-0.12	0.853
rs6577152_TT vs. rs6577152_GG	0.30	0.477	0.09	0.993	0.26	0.077
rs6577152_GT vs. rs6577152_GG	0.52	0.077	0.17	0.198	0.35	**0.035**
